# Estimation of the cutoff value of vitamin D: the Dong-gu study

**DOI:** 10.1186/s40101-015-0048-4

**Published:** 2015-03-12

**Authors:** Seong-Woo Choi, Sun-Seog Kweon, Jin-Su Choi, Jung-Ae Rhee, Young-Hoon Lee, Hae-Sung Nam, Seul-Ki Jeong, Kyeong-Soo Park, So-Yeon Ryu, Hye-Rim Song, Min-Ho Shin

**Affiliations:** Department of Preventive Medicine, Chosun University Medical School, 309, Pilmun-daero, Dong-gu, Gwangju, 501-759 Republic of Korea; Department of Preventive Medicine, Chonnam National University Medical School, 160 Baekseo-ro, Dong-gu, Gwangju, 501-746 Republic of Korea; Jeonnam Regional Cancer Center, Chonnam National University Hwasun Hospital, 322 Seoyang-ro, Hwasun, Jeollanamdo, 519-809 Republic of Korea; Department of Preventive Medicine & Institute of Wonkwang Medical Science, Wonkwang University School of Medicine, 344-2 Shinyong-dong, Iksan, Jeollabukdo, 570-711 Republic of Korea; Department of Preventive Medicine, Chungnam National University Medical School, Munhwa 1(il)-dong,, Jung-gu, Daejeon 301-747, Republic of Korea; Department of Neurology & Research Institute of Clinical Medicine, Biomedical Institute of Chonbuk National University Hospital, Chonbuk National University, San 2-20, Geumam-dong, Deokjin-gu, Jeonju, Jeollabukdo 561-180, Republic of Korea; Department of Preventive Medicine, Seonam University College of Medicine, 439, Chunhyang-ro, Namwon, Jeollabukdo, 590-711 Republic of Korea; Department of Laboratory Medicine, Chonnam National University Hwasun Hospital, 322 Seoyang-ro, Hwasun, Jeollanamdo, 519-809 Republic of Korea

**Keywords:** Vitamin D, Parathyroid hormone, 25-hydroxyvitamin D, Cutoff value

## Abstract

**Background:**

Vitamin D plays an essential role in bone health and growth, but the optimal serum 25-hydroxyvitamin D (25(OH)D) concentration is not known. This study was performed to investigate the optimal 25(OH)D concentration in regard to parathyroid hormone (PTH) concentration in the Korean general population aged 50 years or older.

**Findings:**

The study population consisted of 8,857 subjects (3,545 men and 5,312 women) who participated in the baseline survey of the Dong-gu study, conducted in Korea between 2007 and 2010. Serum 25(OH)D and PTH concentrations were measured by chemiluminescent microparticle immunoassay. The optimal 25(OH)D concentration was estimated by using nonlinear regression model. Our data show that PTH concentration reached a theoretical plateau at 38.2 pg/ml and corresponding 25(OH)D concentration was 21.1 ng/ml in men and PTH concentration at 42.9 pg/ml and 25(OH)D concentration at 13.8 ng/ml in women.

**Conclusions:**

These results indicate that, for Korean general population aged 50 years or older, the optimal 25(OH)D concentration is 21.1 ng/ml in men and 13.8 ng/ml in women.

## Background

Vitamin D deficiency is well known to be a risk factor and an important determinant of osteoporosis [1,2]: 80% to 90% of vitamin D is produced from 7-dehydrocholesterol in the skin after adequate ultraviolet exposure. Only 10% to 20% of vitamin D is derived from dietary sources such as oily fish, milk, butter, eggs, and supplements [3]. Vitamin D maintains circulating calcium levels by regulating ionized calcium absorption in the bone and intestine, and it indirectly affects the concentrations of parathyroid hormone (PTH) [4].

PTH is a regulator of calcium and phosphate homeostasis [5]. Its secretion increases in response to decreased plasma concentrations of calcium, and it acts to elevate circulating calcium levels by promoting the synthesis of active vitamin D (1,25(OH)D) in the kidney. Additionally, PTH stimulates both calcium release from bone and intestinal calcium absorption and increases the reabsorption of active renal calcium [6].

Based on the hypothesis of hypovitaminosis D and secondary hyperparathyroidism, many researchers have investigated the relationship between vitamin D and PTH to find the optimal vitamin D concentration. However, the results were inconsistent [7-12]. Moreover, evidence from the Korean population is limited [13]. The aim of this study was to investigate the optimal 25-hydroxyvitamin D (25(OH)D) concentration in regard to PTH concentration in 50 years or older urban Koreans.

## Methods

### Subjects

The Dong-gu study is an ongoing prospective population-based study that was designed to investigate the prevalence, incidence, and risk of factors for chronic disease in an urban elderly population [14]. It enrolled 9,260 subjects (3,711 men and 5,549 women) aged 50 years or older between April and July in 2007 to 2010 in the Dong-gu district of Gwangju Metropolitan City in Korea (35° N). After exclusion of 347 participants who had incomplete data and 56 participants with estimated glomerular filtration rate (eGFR) values of <30 ml/min/1.73 m^2^, a total of 8,857 subjects (3,545 men and 5,312 women) were included in the present analyses. All participants provided informed consent, and the study was conducted in accordance with the guidelines in the Declaration of Helsinki. The study was approved by the Institutional Review Board of Chonnam National University Hospital (IRB no. I-2008-05-056).

### Measurements

Trained examiners interviewed patients using a standardized questionnaire that assessed cigarette use, alcohol consumption, physical activity, and menopausal status. Blood was drawn from an antecubital vein after a 12-h overnight fast. Serum was separated within 30 min and stored at −70°C until analysis. All samples were measured using an automated analyzer (model 7600 chemical analyzer, Hitachi Ltd., Tokyo, Japan). The concentrations of serum 25 (OH)D and PTH were measured using an ARCHITECT i2000 chemiluminescent microparticle immunoassay analyzer (Abbott Diagnostics, Abbott Park, IL, USA). The coefficient of variation for the total analytic precision of the assay was ≤10% for 25(OH)D and ≤9% for PTH. The lower detection limit of the assay was 3.0 ng/ml for 25(OH)D and 1.0 pg/ml for PTH.

### Statistical analysis

Data are presented as the mean ± standard deviation (SD), or as percentages for categorical variables Vitamin D deficiency was defined as 25(OH)D concentration <20 ng/ml. The statistical analysis was conducted using SPSS 18.0 (SPSS, Chicago, IL, USA). The scatterplot and nonlinear regression analysis were used to examine the association between the serum PTH and 25(OH)D concentrations for possible thresholds.

## Results and discussion

The mean age of the subjects was 65.1 ± 8.1 years, and 60.0% were female. The mean 25(OH)D concentration was 19.2 ± 5.9 ng/ml for males and 15.0 ± 5.1 ng/ml for females (*P* < 0.001). The mean PTH concentration was 40.6 ± 17.7 pg/ml for males and 44.0 ± 19.4 pg/ml for females (*P* < 0.001). Male subjects were more likely to be older, have a lower body mass index (BMI), smoke, drink alcohol, and be physically active. The prevalence of vitamin D deficiency ( <20 ng/ml) was 59.8% in men and 86.2% in women (Table 1).

**Table 1:** **Baseline characteristics of subjects**

	**Male**	**Female**	**Total**	***P*** **values**
*N* (%)	3,545 (40.0)	5,312 (60.0)	8,857 (100.0)	-
Age (years)	66.1 ± 8.0	64.4 ± 8.2	65.1 ± 8.1	<0.001
BMI (kg/m^2^)	23.9 ± 2.8	24.6 ± 3.0	24.3 ± 2.9	<0.001
Month of blood collection				0.004
April	762 (21.5)	1,010 (19.0)	1,772 (20.0)	
May	1,132 (31.9)	1,749 (32.9)	2,881 (32.5)	
June	1,079 (30.4)	1,580 (29.7)	2,659 (30.0)	
July	572 (16.1)	973 (18.3)	1,545 (17.4)	
Smoking (%)	865 (24.4)	99 (1.9)	964 (10.9)	<0.001
Alcohol intake (%)	2,416 (68.2)	1,690 (31.8)	4,106 (46.4)	<0.001
Physically active^a^ (%)	920 (26.8)	667 (13.0)	1,587 (18.5)	<0.001
PTH (pg/ml)	40.6 ± 17.7	44.0 ± 19.4	42.7 ± 18.8	<0.001
25(OH)D (ng/ml)	19.2 ± 5.9	15.0 ± 5.1	16.7 ± 5.8	<0.001
Vitamin D deficient^b^ (%)	2,120 (59.8)	4,595 (86.5)	6,715 (75.8)	<0.001
Postmenopause (%)		5,081 (96.2)		

All values are given as *N* (%) or mean ± standard deviation. BMI, body mass index; 25(OH)D, 25-hydroxyvitamin D; PTH, parathyroid hormone. ^a^Subjects who performed 30 min or more of moderate activity at least 5 days a week or 20 min of vigorous physical activity at least 3 days a week were regarded as doing physical activity; ^b^25(OH)D <20 ng/ml.

The relationship between serum 25(OH)D and PTH concentrations was studied using the nonlinear regression model [9,15]:

$$ \mathrm{P}\mathrm{T}\mathrm{H}\ \left(\mathrm{pg}/\mathrm{ml}\right)=\mathrm{a}+b\times \exp \left(c\times 25\left[\mathrm{O}\mathrm{H}\right]\mathrm{D}\ \left[\mathrm{ng}/\mathrm{ml}\right]\right). $$

Our resulting equations were as follows:

$$ \mathrm{P}\mathrm{T}\mathrm{H}\ \left(\mathrm{pg}/\mathrm{ml}\right)=34.94 + 65.24\times \exp \left(-0.142\times 25\left[\mathrm{O}\mathrm{H}\right]\mathrm{D}\ \left[\mathrm{ng}/\mathrm{ml}\right]\right)\ \left(\mathrm{Male}\right) $$

$$ \mathrm{P}\mathrm{T}\mathrm{H}\ \left(\mathrm{pg}/\mathrm{ml}\right)=38.82 + 86.81\times \exp \left(-0.215\times 25\left[\mathrm{O}\mathrm{H}\right]\mathrm{D}\ \left[\mathrm{ng}/\mathrm{ml}\right]\right)\ \left(\mathrm{Female}\right) $$

**Figure 1:**
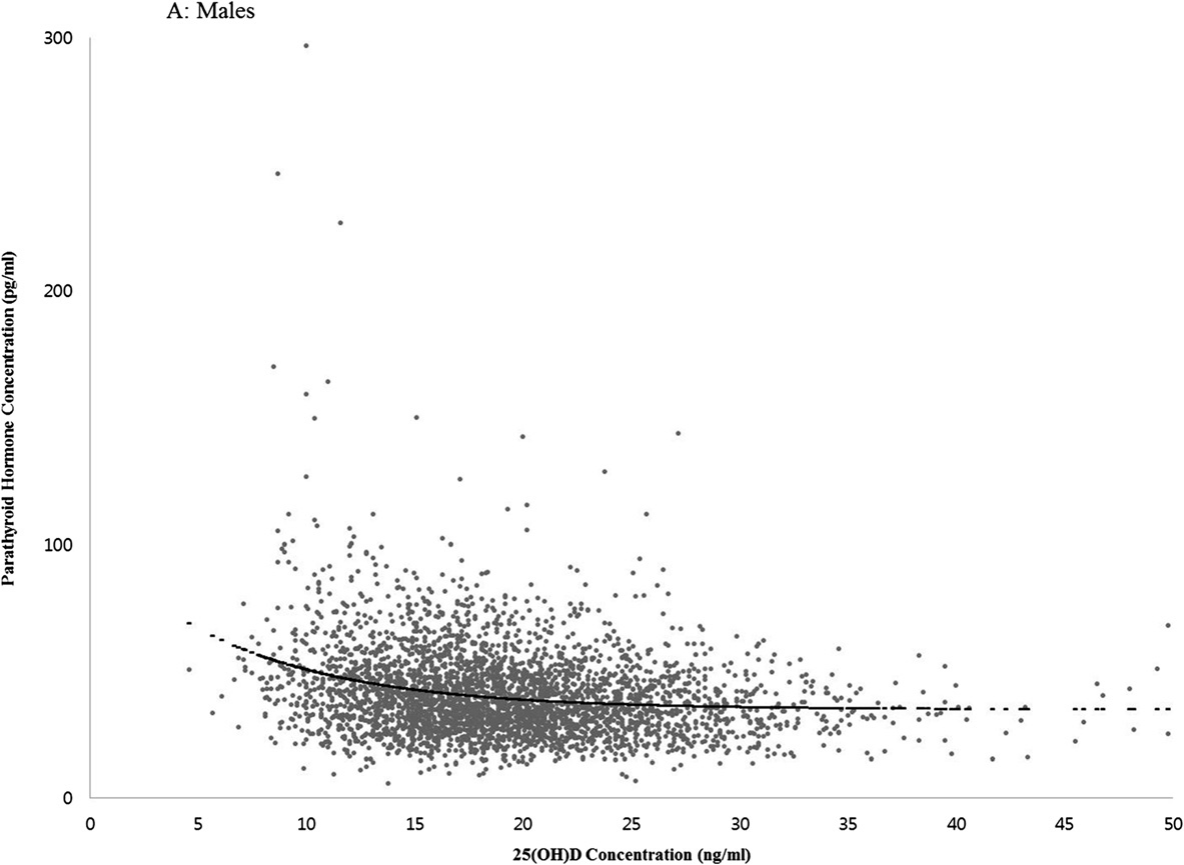
**25(OH)D concentration** ***versus*** **parathyroid hormone concentration (males).**

**Figure 2:**
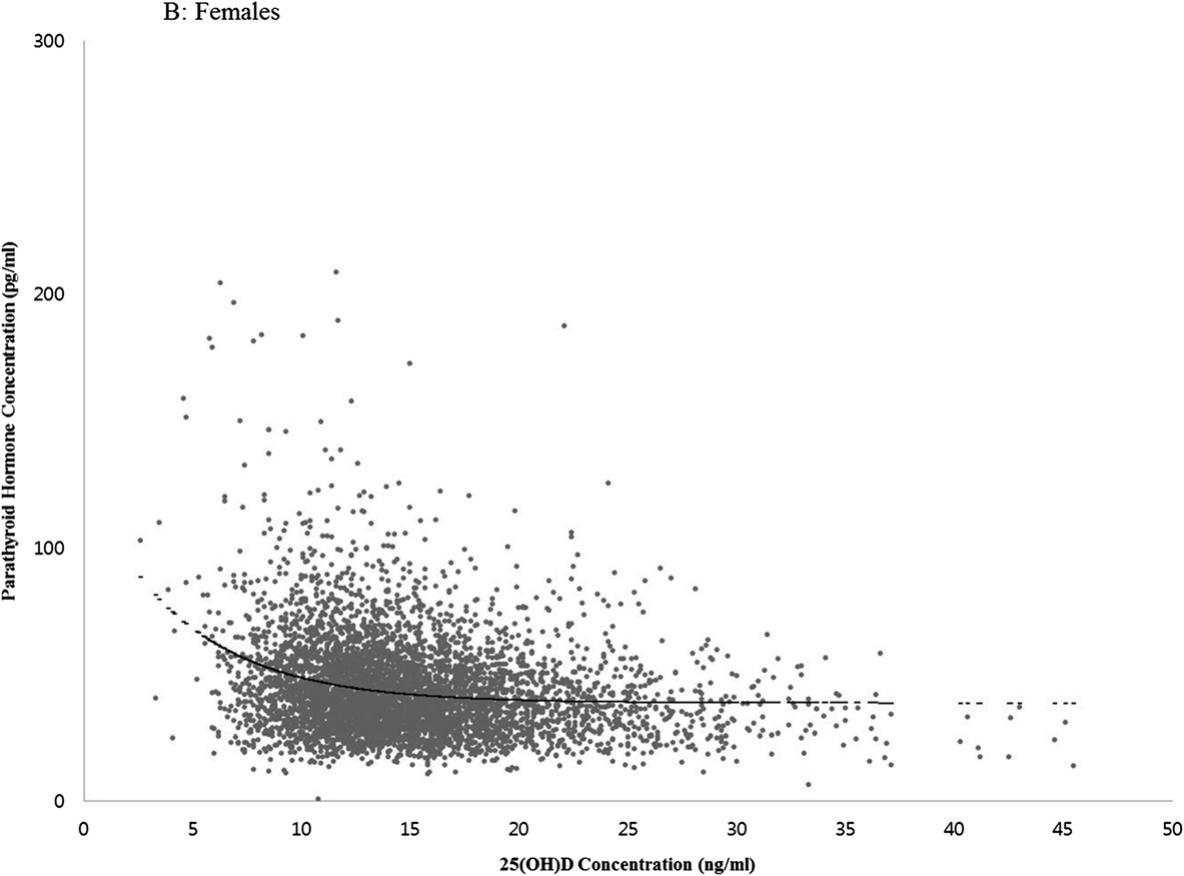
**25(OH)D concentration** ***versus*** **parathyroid hormone concentration (females).**

With this approach, the PTH concentration reached a theoretical plateau at 38.2 pg/ml and the corresponding 25(OH)D concentration was 21.1 ng/ml in men (Figure 1) and the PTH concentration at 42.9 pg/ml and the 25(OH)D concentration at 13.8 ng/ml in women (Figure 2).

The optimal 25(OH)D concentration, the threshold value for 25(OH)D at which PTH plateaus, has been suggested based on the inverse relation between 25(OH)D and PTH [16]. Because PTH is negatively associated with greater bone loss, maintaining a sufficient concentration of 25(OH)D is believed to have a protective effect on bone health [16]. However, there is a lack of consensus as to what constitutes an optimal 25(OH)D concentration. Some researchers reported that an optimal threshold value was not found [17], while others reported a wide range of estimates: 8 to 44 ng/ml [7-12], with most clustered at 30 to 44 ng/ml [9-12]. In the present study, PTH reached a plateau at a 25(OH)D concentration of 30 to 40 ng/ml in males, but did not reach a plateau in females. It is possible that ethnic differences may have influenced the relationship between 25(OH)D concentrations and PTH. In the NHANES study [18], the optimal concentration was 20 ng/ml in Black Americans but was not found in White or Mexican Americans.

Our data demonstrate the optimal concentration was different according to gender. 25(OH)D is well known to be affected by gender differences. Men tend to spend a greater amount of time outdoors than women [19], and the difference in sun exposure may play a role in gender-specific 25(OH)D concentrations. 25(OH)D-binding protein (DBP) may also contribute to gender differences in 25(OH)D status [20], as DBP levels are significantly higher in women than in men and are positively correlated with overall 25(OH)D concentrations [20].

The ongoing difficulties and controversies associated with the relationship between PTH and 25(OH)D imply that this approach is not the best one to identify vitamin D sufficiency in populations. New approaches to this problem, potentially beyond the hypovitaminosis D, secondary hyperparathyroidism pathway, should be pursued [16].

The main strengths of this study lie in its population-based design and use of a relatively large sample size, which minimized selection bias and provided sufficient statistical power. However, a number of limitations should also be considered. First, the study used a cross-sectional design. Second, it had a comparatively limited ability to explain seasonal changes in 25(OH)D, partly due to a lack of information on sun exposure during the four seasons because the samples were collected April to June during 2007 to 2010. Finally, we performed only a single measurement of the serum 25(OH)D concentrations; therefore, the data reflect only a single point in time rather than long-term exposure.

## Conclusion

We estimated the optimal 25(OH)D concentration in the Korean general population aged 50 years or older. The optimal 25(OH)D concentration is 21.1 ng/ml at 38.2 pg/ml PTH concentration in men and 13.8 ng/ml 25(OH)D concentration at 42.9 pg/ml PTH concentration in women.

## Abbreviations

25(OH)D: 25-hydroxyvitamin D
BMI: body mass index
eGFR: estimated glomerular filtration rate
LOWESS: locally weighted estimated scatterplot smoothing
PTH: parathyroid hormone
